# Concentrated transdiagnostic and cross-disciplinary group treatment for patients with depression and with anxiety: a pilot study

**DOI:** 10.1186/s12888-022-04229-y

**Published:** 2022-09-05

**Authors:** Gerd Kvale, Ane Wilhelmsen-Langeland, Marte Jürgensen, Sigurd William Hystad, Lars-Göran Öst, Eirik Søfteland, Tore Børtveit

**Affiliations:** 1 Haukeland University Hospital, Bergen Division of Mental Health, 5021 Bergen, Norway; 2 Department of Clinical Psychology, University of Bergen, Bergen, Norway; 3 Helse i Hardanger, Øystese, Norway; 4 Department of Psychosocial Science, University of Bergen, Bergen, Norway; 5 Department of Psychology, Stockholm University, Stockholm, Sweden; 6 Department of Medicine, Haukeland University Hospital, Bergen, Norway; 7 Department of Clinical Science, University of Bergen, Norway, Norway; 8 Division of Mental Health and Addiction, Vestfold Hospital, Vestfold, Norway

**Keywords:** Depression, Anxiety, Concentrated, Group treatment, Interdisciplinary

## Abstract

**Background:**

A number of treatment approaches have shown efficacy for depression and/or anxiety, yet there is a paucity of research on potentially cost-effective concentrated approaches. Based on our previous experience with concentrated treatment in disorders such as Obsessive–Compulsive Disorder and chronic fatigue, we proposed that this novel approach could be useful for other conditions, including depression and/or anxiety. As a pre-requisite for a future randomized controlled trial, the aim of this study was to investigate the acceptability, satisfaction and effectiveness of a transdiagnostic, interdisciplinary group treatment delivered during 5 consecutive days to groups of 6–10 patients with depression and/or anxiety.

**Methods:**

This was a non-randomized clinical intervention pilot study in line with a published protocol. Forty-two consecutively referred patients, aged 19–47 (mean age 31.7, *SD* = 8.12) were included and completed treatment. All had a severity of their problems that entitled them to care in the specialist public mental health care. Self-reported age when the symptoms became a problem was 20.9 years. Mean number of prior treatment courses was 2.77 (*SD* = 2.19; range 0–8). Acceptability was defined as the proportion of eligible patients who accepted and completed the treatment. Satisfaction was evaluated by Client Satisfaction Questionnaire-8. Secondary objectives were to assess the treatment effectiveness by questionnaires at pre-treatment, seven days post-treatment and three months follow-up.

**Results:**

The treatment was highly acceptable (91.3% accepted, all completed), and patients were highly satisfied with the treatment, including the amount. Functional impairment, as measured by Work and Social Adjustment Scale (WSAS) improved significantly (*p* < .0005) from “severe” (mean 25.4 *SD* = 6.59) to “less severe” (mean 13.37, *SD* = 9.43) at 3 months follow-up. Using the Generalized Anxiety Disorder Scale (GAD-7) and the Patient Health Questionnaire (PHQ-9), the effect sizes at 3 months follow-up were 1.21 for anxiety and 1.3 for depression. More than 80% reported reduced utilization of mental health care, and 67% had not used, or had used the family doctor less, for anxiety or depression. 52% had not used, or had reduced, medication for their disorder.

**Conclusions:**

The concentrated, interdisciplinary treatment approach yielded promising results. Long-term follow up is warranted.

**Trial registration:**

This study is registered in Clinical Trials, identifier NCT05234281 and approval date 09/02/2022.

## Introduction

Patients with depression and anxiety represent the largest group and have the longest treatment courses in the outpatient specialist mental health care [1]. These serious challenges typically have an onset before the age of 25, and significantly affect most aspects of functioning, including the ability to work. In spite of current treatment approaches, anxiety and depressive disorders lead to more lost working years than other major causes of disability (such as musculoskeletal disorders), as those receiving such disability pension are typically younger when receiving it [2]. Given the prevalence and societal burden of these disorders, further development of concentrated and cost-effective treatments is highly warranted [3–5].

Previously, we have documented the effectiveness of 4-day group treatments for patients suffering from chronic fatigue syndrome (CFS-ME; [6]), for obsessive compulsive disorder (OCD; [7, 8]) and for panic disorder (PD;[9]). Our previous results have substantially increased our knowledge base on how care can be delivered to these groups of patients. Hence, we proceeded to evaluate this novel approach in other conditions, and have now developed a comprehensive transdiagnostic concentrated treatment protocol for a broad range of chronic health challenges (anxiety and depression, chronic low back pain, post-covid 19 symptoms, and type 2 diabetes; [10]). These conditions represent a huge burden on our society and our ability to deliver adequate health care, and treatment approaches that can be useful across this heterogeneity are highly warranted. One of the main transdiagnostic features is a shift from monitoring of symptoms to identify moments where the patient is encouraged to systematically break unhelpful patterns of symptom regulation (micro-choices). In order to increase potential cost-effectiveness, the intervention was delivered in a group setting. Furthermore, this provided participants with a relevant peer group when testing out novel approaches to handle their symptoms [6, 7]. For further elaboration, see the protocol paper [10].

We now report the findings in patients with depression and/or anxiety, whereas the somatic illnesses will be collectively reported later due to temporal differences in inclusion rates induced by the COVID pandemic. The goal of the intervention was to enable the participants to live a life where the symptoms of anxiety and depression do not limit or restrict everyday life. Thus, a focus on establishing and maintaining a daily rhythm for sleep-wakefulness, regular meals and activity, which might be compatible with having a job, was integrated throughout the treatment. The intervention explicitly targets a change in symptom regulation, and of how to deal with worry and rumination based on a pragmatic selection of elements from a number of treatment approaches such as cognitive behavioral therapy (CBT) the metacognitive model [11–13]; behavior activation [14] and Acceptance and commitment therapy (ACT; [15]).

In the current paper we report the initial results from concentrated group treatment for patients suffering from depression and/or anxiety, and the aim of this pilot study was to: 1) Explore the acceptability of the intervention measured by the proportion of the patients who met the inclusion criteria, who also accepted participation, and the proportion of included patients who attended the concentrated treatment and finally completed it. 2) Explore the patients` satisfaction with the treatment using the Client Satisfaction Questionnaire (CSQ-8; [16]). 3) Explore potential improvements in level of functioning measured by the Work and Social Adjustment Scale (WSAS; [17]), reduction in anxiety measured by (GAD-7; [18]), and depression measured by (PHQ-9; [19]). Further, we evaluated changes to the illness perception, as described by the Brief Illness Perception Questionnaire (BIPQ; [20]). We also wanted to explore the participants’ utilization of health care as well as potential changes in psychotropic medication, measured by self-report.

Based on our experiences with other concentrated treatment formats [6, 7, 21, 22], we expected ≥ 90% of patients who met the inclusion criteria to accept participation, and ≥ 90% of included patients to attend the concentrated intervention and complete it. We also expected clinically relevant effects on symptoms of anxiety and depression as well as significant improvement in functional impairment. We have not previously reported on changes in utilization of health care, but expected these to be unchanged or reduced.

## Method

This pilot study is part of the “Project Development of Smarter Health Solutions” (PUSH project), a collaboration between Haukeland University Hospital (Bergen, Norway) and Helse i Hardanger (HiH; Øystese, Norway). The overall aim, as well as the organization of the PUSH project, is described in a separate protocol paper [10]. Further, to ensure adherence and fidelity, all study-related procedures were operationalized in a Standard Operating Procedure (SOP). Each group was allocated with a psychologist whose task was to ensure protocol adherence. All plenary sessions were audiotaped and checked for competency and fidelity by the first author and no deviances were found.

### Organization of the project: training and staffing

HiH delivers concentrated treatment to patients with low-back pain, diabetes type 2, post Covid-19 fatigue symptoms as well as to patients with anxiety and depression [10]. The main architects behind the content of the program for patients with depression and/or anxiety are the first and last author of the current article (GK and TB). The study therapists were psychologists or psychiatrists with extensive training and experience (> 15 years). All participating clinicians have received hands-on training and supervision from the first or last author during screening and the pre-treatment preparation. All parts of the treatment, as well as the screening and inclusion procedure, the pre-treatment preparation, and the patients’ continued work during the first three months post-treatment are manualized. The two group leaders allocated full time to the group throughout the week, and did not combine this with other tasks. Other members of the interdisciplinary team, like the physiotherapist or the clinical dietitian participated in specific sessions, and were updated on the progress and challenges for each patient. Furthermore, a pharmacist was a member of the team, and available if needed.

### Referral procedures

General practitioners in the uptake area were encouraged to refer patients to the project. If the patients’ symptoms after initial evaluation according to the Norwegian priority guidelines suggested a condition that was severe enough to grant them treatment as a part of specialist public health care (moderate to severe mental illness or conditions that is expected to improve substantially with treatment offered), they were screened for participation in the project via a structured short telephone-interview within 10 days after referral (typically lasting 10 min) [23]. They then had two consultations with a clinical psychologist, either face-to-face or via a secure online platform. Most patients had the first consultation within 4 weeks from referral, in accordance with national priority guidelines. Time-slots for groups were decided in advance, and eligible patients were offered participation successively upon availability. Waiting times (1–10 weeks) were in line with the priority guidelines in Norway.

### Patients

#### Inclusion and exclusion criteria

Patients between 18 and 47 years of age were eligible for inclusion if they fulfilled the ICD-10 [24] criteria for one of the following disorders: depression (F33.0–3); generalized anxiety disorder (F 41.1); mixed anxiety and depressive disorder (F 41.2); other mixed anxiety disorders (F 41.3) or unspecified anxiety disorder (F 41.9), and this being their main psychiatric problem. Patients with a principal diagnosis of obsessive–compulsive disorder (F 42), panic disorder with or without agoraphobia (F41.0/F40.0), social anxiety disorder (F40.1) or chronic fatigue (G93.3 or F48.0) were excluded since patients with these disorders already had adequate concentrated treatment opportunities within the catchment area. The participants needed to be fluent in Norwegian.

Exclusion criteria were bipolar disorder, psychosis, ongoing severe or primary substance abuse/dependence, mental retardation based on previous medical history, very low BMI in need of medical attention, and ongoing suicidal ideation. Also, if the patients had a physical condition which prevented them from participating in physical exercises or training, they were not offered participation.

**Fig. 1:**
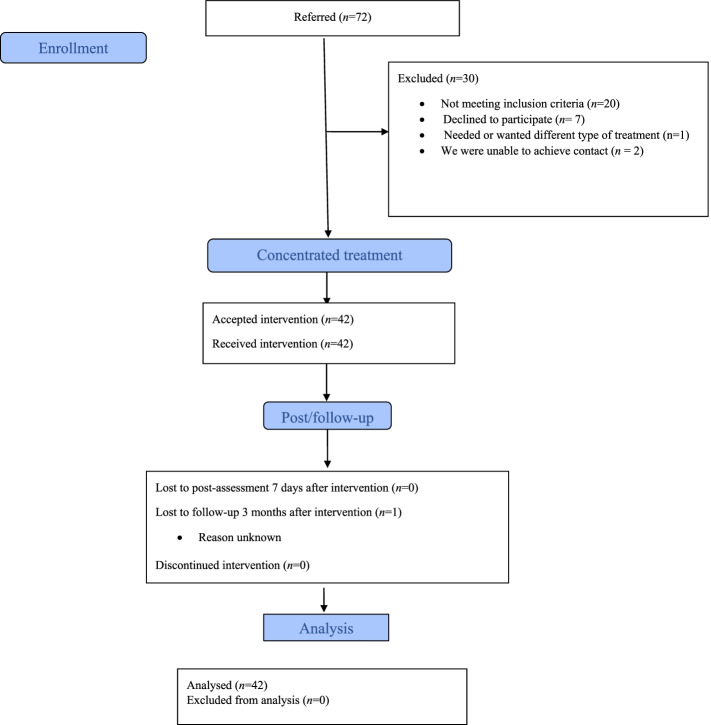
Flow-chart showing patient flow through the project

During the inclusion period, 72 patients were referred and 20 were excluded as they did not fulfil the inclusion criteria. Three declined to participate as they were ambivalent about the concentrated group format, and were not further evaluated, one was in need of a different type of treatment with longer follow-up, and finally we were unable to get in contact with two of the referred patients. Of 46 patients who fulfilled the inclusion criteria, 42 accepted (91%) (see Fig. 1 for flow chart).

##### Background information

Table 1 displays a summary of background data for the sample.

**Table 1:** Background information on patient sample (*n* = 42)

	*M (SD)*	*n*	*n (%)*
**Gender**			
Female		22	52.4
**Age**	31.7 (8.12)		
**Age at onset of problems**	17.4 (7.34)		
Duration of problems	14.2 (9.31)		
**Previous treatment courses**	2.77 (2.12)		
None		4	9.5
One		9	21.4
Two or more		29	69.1
**Marital status**			
Single		21	50
Married or cohabitating		20	47.6
Unknown		1	2.4
**Educational status**			
Junior high school		3	7.1
High school		17	40.5
College		17	40.4
Apprenticeship		5	11.9
**Work status**^a^			
Employed		28	66.7
Unemployed		4	9.5
Job applicant		1	2.4
College student		1	2.4
High school student		2	4.8
Unknown/other		6	14.2
**Months out of work**	9.61 (2.85)		

^a^ Missing info from two participants regarding work status

In total 90.5% of the patients had received previous treatment courses (specialist health care, primary/community health care, private sector) for the current disorders. The majority of the patients (59.5%) reported that they used prescribed medication for depression and/or anxiety. No changes of medication were mandated by participation in the project.

Assessment of comorbidities, including sleep difficulties, hypochondriasis, attention deficit hyperactivity disorder (ADHD/ADD), neurasthenia and post-viral fatigue was based on referral reports. Thirty-nine (92.9%) reported sleep difficulties, 4.8% had hypochondriasis, and 2.4% had diagnosed ADHD. Only 7.1% of the included patients had no current comorbidity.

### Assessment

#### Outcomes

The primary objective of this study was to evaluate the acceptability and satisfaction with the treatment format. Secondary objectives were to assess changes to patients’ symptoms and level of functioning through questionnaires at pre-treatment, seven days post-treatment (not level of functioning) and three months follow-up. Further explorative end-points were changes to utilization of health care, and to the use of psychotropic medications.

#### Acceptability

Acceptability was defined as the rates of eligible patients who accepted participation, as well as the rates of included patients who completed the treatment.

#### Diagnostic interview

Before treatment started, the patients went through a diagnostic interview by a trained psychologist using the Mini International Neuropsychiatric Interview (M.I.N.I.; [25]). M.I.N.I is a short structured diagnostic interview, which screens axis-1 DSM-IV disorders, and the Norwegian version has good psychometric properties [26].

#### Questionnaires

Patients answered standardized self-report questionnaires online. The questionnaires covered symptoms of anxiety, depression, functional impairment as well as utilization of health care and use of psychotropic medication at three-month follow up. If patients did not complete self-report questionnaires according to a pre-set time limit, an automated text message was sent to their phones.

##### Treatment satisfaction

In order to obtain a measure of the patients’ degree of satisfaction with the treatment the Client Satisfaction Questionnaire (CSQ-8 [27]; was applied one week after the concentrated treatment. This is an 8-item questionnaire that measures patient satisfaction with health services, where the items are rated from 1 (very low satisfaction) to 4 (very high satisfaction). The total score ranges from 8–32, with higher scores indicating higher degree of satisfaction. The CSQ-8 has good psychometric properties, with high internal consistency (Cronbach’s α = 0.93), and high inter-item correlation [27]. Cronbach’s α in the current study was 0.83.

Functional level was assessed pre-treatment and at 3-month follow-up by The Work and Social Adjustment Scale (WSAS; [28]) which is a short questionnaire measuring the impact of the disorder on aspects of work and social activities. The scale consists of five items rated from 0 (not at all) to 8 (very severe), and a higher score indicates higher impairment (maximum score is 40). The cut-off for moderately severe or worse psychopathology is > 20, scores between 10–20 are associated with significant functional impairment but less severe clinical symptomatology, and scores < 10 are associated with subclinical populations. The scale has good psychometric properties [28]. Cronbach’s α in the current study were 0.64 at baseline and 0.90 at 3-months follow-up.

Depressive symptoms were measured at pre-treatment, post-treatment and follow-up, by The Patient Health Questionnaire-9 (PHQ-9; [19]). The PHQ-9 is a self-report scale with nine items (0 to 3 scale). The maximum score is 27. A score of 10 or more is indicative of a depressive disorder [19]. Recently the cut-off value was suggested to be 14 or higher [29]. Cronbach’s α in the current study were 0.69 at baseline, 0.81 at post-treatment, and 0.86 at 3-months follow-up.

Symptoms of generalized anxiety was measured at pre-treatment, post-treatment and follow-up by The Generalized Anxiety Disorder Scale (GAD-7; [18]. The GAD-7 is a self-report scale with seven items (0 to 3 scale). The maximum score is 21 and the suggested cut-off of is 10 points. Cronbach’s α in the current study were 0.81 at baseline, 0.79 at post-treatment, and 0.87 at 3-months follow-up.

Illness perception was measured at pre-treatment, post-treatment and follow-up by the Brief Illness Perception Questionnaire (BIPQ), which is a 9-item questionnaire designed to assess cognitive and emotional representations of illness [20]. Five of the BIPQ items assess cognitive representations (consequences, timeline, personal control, treatment control, and identity), two of the items assess emotional representations (concern and emotions), and one item assesses illness coherence. These eight items were summed together to calculate a composite BIPQ score (α = 0.52 at pre-treatment, α = 0.84 at post-treatment, and α = 0.82 at follow-up). The personal control, treatment control and coherence items were reversed prior to computing the composite score. A higher BIPQ score indicates a greater perceived psychological burden of illness. Questions are graded from 0 to 10. The last item deals with the perceived cause of illness, in which respondents list the perceived three most important causal factors in their illness. The scale has good psychometric properties according to a recent review [30].

##### Clinically relevant evaluation of change

Since our sample is composed of patients with several depression/anxiety disorders, neither PHQ-9 nor GAD-7 would be relevant for all patients. Also, due to limited number of participants, further sub-group analyses were not feasible. However, based on our clinical experience, we included a question addressing overall change in their relevant symptoms. The question was tested out on a smaller sample of patients who reported that they had no problems understanding and assessing this. Thus, three months after treatment, the patients reported on the following questions: 1. “In all, how would you evaluate your anxiety and depression now, compared to before the treatment?” Answers could be a) substantial improvement, b) improvement, c) minimal improvement, d) no change, e) minimal deterioration, f) deterioration, g) substantial deterioration. We have collapsed a-b into “Improvement”, c-e “into “No change” and f-g into “Deterioration” in the results section.

##### Utilization of health care

We also wanted to get feedback from the patients regarding their utilization of health care and the following question was asked three months after the concentrated treatment: “Compared to before the treatment initiated, how has your utilization of health services directly related to anxiety and depression been?” Health services listed were specialist health care, community services, private health services, and general practitioner. Answers could be a) no use, b) less use than before, c) unchanged, and d) more use than before.

##### **Changes in psychotropic medication**

Three months after treatment, the patients answered the following question regarding use of psychotropic medication: “Compared to the start of the treatment, how has your use of medication related to anxiety and depression been?” Answers could be a) less use, b) unchanged, c) more and d) not applicable.

### Procedure

For an overall detailed description of the procedure and the intervention, please refer to the protocol paper [10]. The M.I.N.I. interview [25] was conducted prior to inclusion. If the patient fulfilled the inclusion criteria and none of the exclusion criteria, a video explaining the concentrated treatment was shown (https://youtu.be/fyo9uTzucRM) and subsequently the therapist answered any questions the patient may have.

In order to ensure that the patients were prepared for what to expect, a thorough description of the treatment was presented, delivered in a dialogue fashion where the therapist waited for affirmation of understanding from the patient. The presentation lasted approximately 10 min.

At the end of the screening and information an adapted version of the Borkovec and Nau [31] Reaction to treatment scale was used to assess treatment expectations and treatment credibility. The patient was asked to evaluate four aspects on a 0–100% scale, and if a number below 70% was reported, it served as an opportunity to clarify possible misunderstandings. If the patient wanted to initiate treatment, informed consents were signed. One week prior to treatment, the leader of the group called each patient to ensure they had received all necessary information and were ready. During the concentrated treatment (five days) the patients were discouraged from using anxiolytics and alcohol. No adjustment was made to other concurrent medications. Patients were encouraged to abstain from other treatment for depression and anxiety within three months after the concentrated intervention.

### Treatment

The concentrated treatment took place at an outpatient clinic outside Bergen, and the participants were accommodated at a hotel in the same building. The treatment was delivered to groups of 6–10 patients. The outline and content of the treatment is described in the protocol paper [10]. The treatment was delivered during five consecutive days, starting in the evening (8 pm) the first day, and finishing at lunchtime the last day. The first evening the patients were introduced to the program. Days 2–4 the joint program (830 am to 4 pm) was a composite of education, followed by individual training focused on breaking unuseful patterns of symptom regulation, interspaced with feedback on their performance, delivered in the group sessions. The group was headed by two psychologists, one with substantial experience with concentrated treatment formats. During the week each patient also had one individual session with a psychologist (30 min). During days 2–5 the patients had daily sessions with physical activity. Also, the group had a session with a clinical dietitian. In total, each patient participated in 14 h of group sessions headed by psychologists, five hours with physical activity and one hour with dietitian. Day 5 (830 am -12 pm) “lessons learnt” were summarized, and each patient planned their tasks for the next three weeks. The rest of the time, including afternoons until 7 pm, each patient worked on their individually tailored tasks, and presented their progress in the group sessions. During the concentrated treatment, the total amount of health care resources per patient consequently varied between 3.9–6.2 h, according to group size. Ten days after the end of treatment, the program also included a twenty-minute individual phone or video call by the group leader, focused on how to maintain the change.

### Data completion

All included patients completed the treatment program. A total of 42 patients are included in the dataset. Of the 42 study participants, all completed the post-treatment questionnaires one week after treatment, and 41 (97.6%) at the 3-month follow-up.

### Definition of response and remission

A response was defined as a reduction in WSAS from pre-treatment to follow-up that is considered reliable according to Jacobson and Truax’ [32] reliable change index (RCI), i.e., larger than that reasonably expected due to measurement error alone. RCI was calculated using the present sample’s pre-treatment standard deviation (*SD)* and the mean internal consistency over the two assessment points, yielding a reliable change criterion of 9 points.

Remission was defined as a reliable reduction and a follow-up score that falls outside the range of the patient sample’s pre-treatment distribution, where range is defined as extending to two SDs beyond the mean in the direction of functionality. A cut-off score was therefore calculated as the patient samples’ pre-treatment mean minus 2*SD (25.52 – 2*6.56), yielding a score of 12.

Finally, at 3 months, patients answered the following question: “In all, how would you evaluate your anxiety and depression now, compared to before the treatment?”.

### Statistical analyses

One patient was lost to follow-up three months after the intervention. This person’s last observed scores on the dependent variables were used for the subsequent assessment point (i.e., last observation carried forward). Paired measures *t*-test was used to compare WSAS from pre-treatment to 3-month follow-up, while repeated measures ANOVAs (analyses of variance) were used to compare GAD-7, BIPQ and PHQ-9 across the three assessment points (pre-, post-, and 3-month follow-up). Statistically significant effects were followed-up with Bonferroni post-hoc comparisons. Effect sizes of change over time were calculated using Glass´ Δ, with pre-treatment *SD* as denominator. Glass’s Δ is the recommended effect size for intervention studies in which there are reasons to believe that the treatment will influence the standard deviation as well as the mean [33]. All analyses were conducted using Stata version 17.0 [34].

## Results

### Acceptability

Of the 46 eligible patients, 42 accepted participation (91.3%). All of the included patients completed the treatment.

### Treatment satisfaction

All patients reported high satisfaction with the concentrated treatment. A summary of the CSQ-8 scores is displayed in table 2.

**Table 2:** Post-treatment scores on Client Satisfaction Questionnaire-8 (*n* = 42)

Item	Responses			
	1	2	3	4
*1. How would you rate the quality of service you received*	0%	0%	14.3%	85.7%
*2. Did you get the kind of service you wanted*	0%	2.4%	47.6%	50%
*3. To what extend has our program met your needs*	0%	2.4%	38.1%	59.5%
*4. If a friend were in need of similar help, would you recommend our program to him or her?*	0%	0%	4.8%	95.2%
*5. How satisfied are you with the amount of help you received*	0%	0%	33.3%	66.7%
*6. Have the services you received helped you to deal more effectively with your problems?*	0%	4.6%	26.2%	69%
*7. In an overall, general sense, how satisfied are you with the service you have received?*	0%	0%	16.7%	83.3%
*8. If you were to seek help again, would you come back to our program?*	0%	2.4%	19%	78.6%

*Note*. Mean CSQ-8 score was 29.76(SD 2.53), range 22-32, median = 30 (possible range is 8 – 32). Description of responses:

4 = Excellent; Yes, definitely; Almost all my needs have been met; Very satisfied; Yes, they helped a great deal

3 = Good; Yes, generally; Most of my needs have been met; Mostly satisfied; Yes, they have helped somewhat; Yes, I think so

2 = Fair; No, not really; Only a few of my needs have been met; Indifferent or mildly dissatisfied; No, they really did not help

1 = Poor; No, definitely not; None of my needs have been met; Quite dissatisfied; No, they seemed to make things worse

### Clinical outcomes

As expected, patients showed a significant and large improvement in the level of functioning (WSAS) from pre-treatment to 3-month follow-up (ES = 1.86 (see Table 3)). The repeated measures ANOVAs further indicated statistically significant changes in depressive symptoms, *F*(2, 82) = 69.12, *p* < 0.001, illness perceptions, *F*(2, 82) = 149.50, *p* < 0.001, and symptoms of generalized anxiety, *F*(2, 82) = 52.54, *p* < 0.001. Post-hoc analyses showed that depression symptoms decreased from pre-treatment to post-treatment (Δ*M* = -7.21, *t* = -10.05, *p* < 0.001) and from pre-treatment to 3-month follow-up (Δ*M* = -7.40, *t* = -10.31, *p* < 0.001). There was no statistically significant difference between post-treatment and follow-up (Δ*M* = -0.19, *t* = -0.27, *p* = 1).

**Table 3:** Measures of degree of work and social functioning, symptoms of generalized anxiety and symptoms of depression, *N* = 42

Measure	Pre	Post	Follow-up	*t/F*	*p-*value	ES pre-post [95% CI]	ES pre-FU [95% CI]
WSAS	25.52 (6.56)_a_	–	13.33 (9.32)_b_	8.39	< .001	–	1.86 [1.36, 2.36]
GAD-7	13.40 (4.42)_a_	8.05 (3.60)_b_	6.55 (3.79_)b_	52.54	< .001	1.21 [0.81, 1.61]	1.55 [1.10, 2.00]
PHQ-9	15.88 (4.39)_a_	8.67 (4,33)_b_	8.50 (5.00)_b_	68.60	< .001	1.65 [1.18, 2.11]	1.68 [1.21, 2.15]

*Note*. *WSAS* The Work and Social Adjustment Scale, *GAD-7* The Generalized Anxiety Disorder Scale, *PHQ-9* The Patient Health Questionnaire-9, *FU* 3-month follow-up after intervention, *ES* Effect size computed as Glass's Δ = $\frac{{M}_{pre}-{M}_{post}}{{SD}_{pre}}$

Different subscripts within a row indicate statistically significant differences between means

The same picture emerged for generalized anxiety. Generalized anxiety symptoms decreased from pre-treatment to post-treatment (ΔM = -5.36, *t* = -7.62, *p* < 0.001) and from pre-treatment to 3-month follow-up (Δ*M* = -6.86, *t* = -9.75, *p* < 0.001). The difference between post-treatment and follow-up was not statistically different (Δ*M* = -1.5, *t* = -2.13, *p* = 0.11).

For illness perceptions, all pairwise comparisons were statistically significant. Illness perception decreased by -36.38 from pre-treatment to post-treatment (*t* = -13.25, *p* < 0.001), and further decreased by -8.24 from post-treatment to 3-month follow-up (*t* = -3.00, *p* = 0.01). The difference in illness perceptions from pre-treatment to 3-month follow-up was Δ*M* = -44.62 (*t* = -16.25, *p* < 0.001).

### Recovery and remission

The IAPT (Improving Access to Psychological Therapies) evaluation counts participants as clinical cases if they fulfill the criterion for caseness on either GAD-7 (≥ 8) or PHQ-9 (≥ 10) [35]. In order to be counted as recovered, the clinical cases have to score below these criteria on both GAD-7 and PHQ-9. There were 35 out of 42 participants (83.3%) who fulfilled the caseness criteria pre-treatment. At post-treatment 37.1% of these fulfilled the recovery criteria and at follow-up 62.9% did so.

Three months after the treatment, 64.3% of patients had shown a response according to the Jacobson and Truax (1991) criteria (minimum 9 points reduction in WSAS) and 52.4% could be classified as remitted (response and ≤ 12 on WSAS).

Based on the single question addressing overall change in relevant symptoms, 80.9% of the patients reported improvement, 14.3% reported no change, and a single patient reported deterioration (2.4%). One patient (2.4%) did not answer this question.

Three months after treatment, 38.1% of the patients reported a substantial improvement, 42.8% reported improvement, 14.3% reported minimal improvement, and one patient (2.4%) reported deterioration.

### Utilization of health care

On the question: “Compared to before the treatment initiated, how has your utilization of health services directly related to anxiety and depression been?”, most patients reported “not using” or “less use” than before regarding specialist health care, community services and private health services (for details see Table 4). The majority of the participants reported that they had not seen or seen their GP less than before (see Table 4).

**Table 4:** Utilization of health care at 3 month follow-up, *n* = 42

	**No use**	**Less than before**	**No change**	**More than before**
Specialist health care	78.6%	4.8%	11.9%	2.4%
Community services	78.6%	4.8%	11.9%	2.4%
Private health care	83.3%	2.4%	9.5%	0%
General practitioner	38.1%	28.6%	26.2%	4.8%

*Note*. Missing data from one participant

### Change in psychotropic medication

Before treatment, 59.5% reported using medication for anxiety and depression. Of this sub-sample, 32% reported less use at follow-up, 8% reported more us, 48% reported unchanged use of such medication and 12% reported it was not applicable. None of those who reported not using such medication before the treatment, had started using it at 3-month follow-up.

## Discussion

In the current pilot study, we reported the results of a concentrated transdiagnostic interdisciplinary group treatment for anxiety and depression. The patients who were included had a mean duration of their anxiety/depression symptoms of 14 years and these had been problematic for 10 years on average. Ninety-three percent had some comorbid disorder, 76% had received previous unsuccessful courses of treatment, 59% used medication for their anxiety/depression symptoms, and 55% had some type of disability pension or sick leave due to their disorders.

A comparison with the Norwegian Prompt Mental Health Care (PMHC) program [36] showed that the current study had a significantly higher proportion with previous treatments (76 vs. 16%), with psychotropic medication (59 vs. 22%), being single (50 vs. 31%), mean GAD-7 score (13.4 vs. 10.1), and mean PHQ-9 score (15.9 vs. 12.5) before the start of treatment. This indicates that the current sample was more severe than the PMHC-sample, in terms of previous treatment attempts, medical treatment and baseline symptoms.

The cross disciplinary intervention lasted less than a week and included elements from cognitive behavioral therapy, behavioral activation, ACT, metacognitive approaches as well as brief mindfulness exercises, all focused on breaking patterns of unhelpful emotional regulation and introducing an eating-sleeping-moving pattern compatible with having a job.

The intervention was highly acceptable (> 90%) and levels of satisfaction were high. Compared to pre-treatment, clinically meaningful improvements in level of functioning were seen, with correspondingly increased perceived understanding of the health challenges. As expected, the improvements did not significantly deteriorate between post-treatment (7 days) and follow-up (3 months).

The recovery rate using the combined GAD-7 and PHQ-9 criterion was 37.1% in the present study. This is almost comparable with the data reported by Clark et al. [35] across all IAPT-services in England; a mean of 42.9% (range 17.6–54.6%) for 2015 and a mean of 44.4% (range 20.4–58.7%) for 2016, and the PMHC-pilot sites in Norway [36] with a mean of 46%. It is probable that the post-assessment done one week after the end of treatment in our study does not reflect the full treatment effect. During the period after treatment the patients have ample opportunities to experience how they function in various situations. At the 3-month follow-up the recovery rate had increased to 62.9%. Unfortunately, neither the IAPT- nor the PMHC-article present follow-up data.

A recent meta-analysis [37] of 47 studies evaluating the English IAPT approach reported a mean pre-post effect size of 0.96 for GAD-7, 0.93 for PHQ-9, and 0.50 for WSAS. The Norwegian version of IAPT (the PMHC; [36]) reported data for the first 12 pilot sites and found a pre-post effect size of 0.74 on GAD-7 and 0.71 on PHQ-9. Thus, the effects in the present study – 1.21 on GAD-7, 1.65 on PHQ-9, and 1.86 on WSAS – are as good or better. Also, there was no dropout in our study, whereas the attrition rate was 15.5% in the Norwegian PMHC and 28.9% in the English IAPT. One possible explanation is that our group treatment was delivered during five consecutive days, as compared to weekly across 11 weeks in PMHC.

Based on our experiences with concentrated treatment formats for other disorders [6, 7, 21, 22] we had expected that more than 90% of the eligible patients would accept inclusion, which was confirmed. Also, all of the patients were satisfied, and none dropped out of the treatment, which is highly unusual for this complex group of patients. We speculate that a number of factors could impact this result; the comprehensive preparation before treatment, where the patients actively choose to participate, the concentrated group format, as well as the emphasis on how to integrate the changes into everyday living.

Given that 70% of the patients had one or more previous treatment courses, it is interesting to note that 100% were satisfied with the amount of treatment, and all of the included patients would recommend it to a friend with similar problems. One week after the intervention 95% reported that the treatment had helped them to deal with their problems, and at three months follow-up 81% reported overall improvement of their problems. Large effect sizes were obtained on measures of anxiety (GAD7) and depression (PHQ9). The WSAS was used to evaluate level of functioning, and on this, 64% gained a clinical reliable response and 52% were in remission at three months’ follow-up. Also, the patients reported a decrease in the utilization of health care related to their problems, and of psychotropic medication.

Even though the content of the treatment differs substantially from our previous concentrated treatment approaches (see e.g. Chronic Fatigue [6], OCD or PD [9, 22]) the main elements pertaining to the format are common. This especially refers to the pre-treatment preparation as well as the concentrated group format where the focus is to give the patients individually tailored experiences with breaking unhelpful patterns of symptom regulation. We emphasized physical activity and natural bodily rhythms (nutrition and sleep–wake, activity-rest) more in the current treatment approach, hence this pilot study is more interdisciplinary and holistic than previous concentrated treatment approaches. Another aspect that differs compared to our previous concentrated treatment formats is the staffing. In the current treatment, the groups of 6–10 patients were led by two therapists working together with a inter-disciplinary team (staffing varying between 1:5 and 1:3), while the concentrated groups for OCD and for PD are staffed with the same number of therapists as patients (1:1) making it an individual treatment delivered in a group context. The reduced staffing, as compared to the OCD and PD groups, is made possible by our treatment where each patient was made responsible for tailoring the relevant exercises for breaking their unhelpful patterns of emotional regulation.

It is also noteworthy, that the format seems to be efficient across disorders and not dependent upon a team that works together on a daily basis, as long as the team members have received proper training and follow the described procedures. This increases the flexibility of the treatment, but it is of utmost importance to ensure that the integrity of the treatment is intact. In the current pilot study, all group sessions were audiotaped and checked for competency and fidelity by the first author, and no deviances were found.

Based on our clinical experiences, the concentrated treatment format seems to work across disorders and can also be used in a transdiagnostic manner, not only for homogenous and diagnosis-specific groups of patients. This might be of relevance when it comes to future implementations.

### Strengths and limitations of the pilot study

This pilot study represents a novel concentrated multidisciplinary approach to treat a heterogeneous group of patients with previous unsuccessful treatments, and with a long history of detrimental symptoms of anxiety and depression, presenting a high risk of prolonged disability and a high burden of disease. The approach implies a concentrated input of resources relative to treatment as usual for depression and anxiety in the public health care. The study shows that patients with long-standing depression and/or anxiety, and with substantial prior treatment experiences, could benefit from a different and more concentrated treatment format. As mental health problems are on the rise, developing effective treatments is crucial for the public health care. In particular, pragmatic studies conducted in a mental health setting – as the current one – are warranted. It should be noted, that the total amount of health care resources per patient in this pilot project was remarkably low (between 2.5–3.8 h), strengthening the potential relevance of concentrated treatment approaches. A further strength of the study was the relatively equal gender distribution, increasing external validity.

Limitations of the study include the short duration, moderate sample size, the relatively experienced group leaders, the fact that there is no control group, and that it is not possible to ascertain or disentangle specifically what are the effective components of the treatment. Due to the diversity and numbers of participants, sub-group analyses were not regarded as feasible. Also, the results are not generalizable to patients who fall outside of the inclusion/exclusion criteria. In particular, this could be relevant for patients aged above 47 years, those lacking digital competency, or patients with other co-morbidities that precluded participation. Further, although all group sessions were audited by a psychologist, to ensure adherence to the protocol, no formal evaluation on competency was performed. Finally, data on the utilization of health care and changes in medication may have limited robustness, since these were based on self-report. Future research into which interventions are the most potent for change is warranted.

## Conclusion

The above described concentrated andinterdisciplinary treatment approach for patients with depression and/or anxiety yielded promising results and cost-effectiveness, with high acceptability, high levels of patient satisfaction and significantly improved levels of functioning at 3-month follow-up. Furthermore, positive changes in depressive and anxiety symptoms were observed during the study. We conclude that these preliminary findings need confirmation in robust research designs including a control group and large sample allowing for relevant subgroup analysis (e.g. diagnosis) as well as treatment and process-variable analysis.

## Data Availability

The data that support the findings of this study are available from Youwell A/S, but restrictions apply to the availability of these data, which were used under license for the current study, and so are not publicly available. Data are however available from the authors upon reasonable request and with permission of Youwell A/S.
